# Supplementary material for: Use of Measurable Residual Disease to Evolve Transplant Policy in Acute Myeloid Leukemia: A 20-Year Monocentric Observation
Source: Cancers (Basel). 2021 Mar 3;13(5):1083. doi: 10.3390/cancers13051083 (PMC7959451; doi:10.3390/cancers13051083)
Supplement: Supplementary file 1 [file cancers-13-01083-s001.pdf]

Buccisano et al., Supplemental materials

Figure 1S.

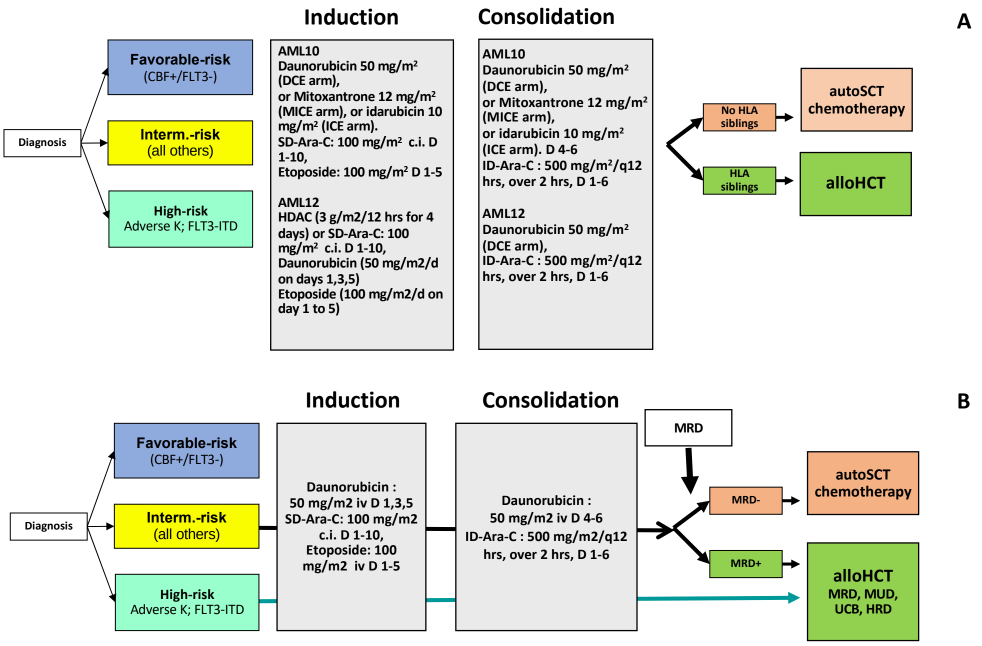

Figure S1. In the donor-driven cohort (A), per AML10/AML12 protocols, transplantation assignment relied only on the availability/lack of a familiar sibling donor, regardless of the individual risk profile. In the MRD-driven cohort (B) post-consolidation assignment was risk-stratified according to the MRD levels for favorable- and intermediate-risk patients.

Figure 2S

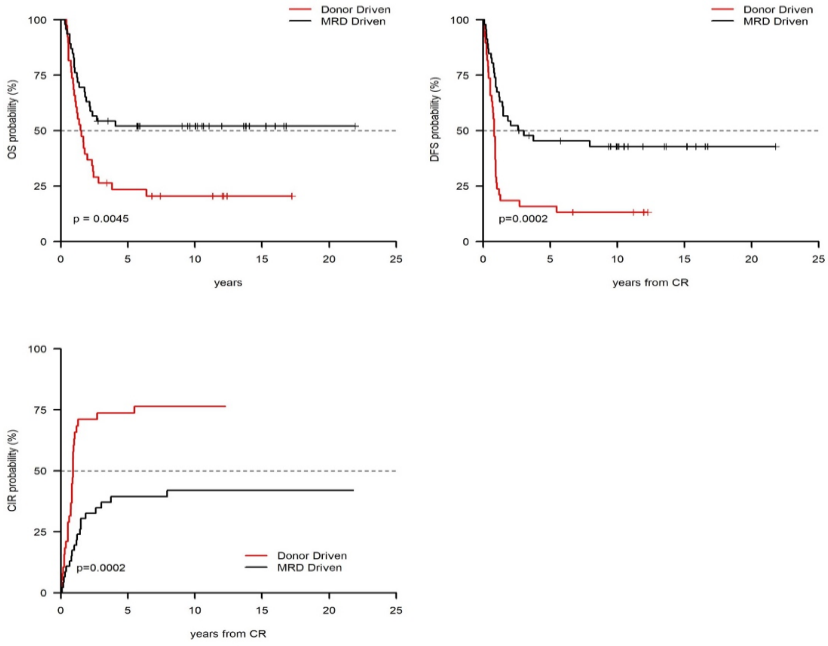

Figure S2. Impact of MRD-driven strategy on Intermediate risk (IR) patients. OS (52.0% vs. 20.5%, p=0.0045), DFS (42.8% vs. 13.2%, p=0.0002) and CIR (42.0% vs. 76.3%, p=0.0002) were significantly better for the MRD-driven cohort than the Donor-driven one.

Figure 3S

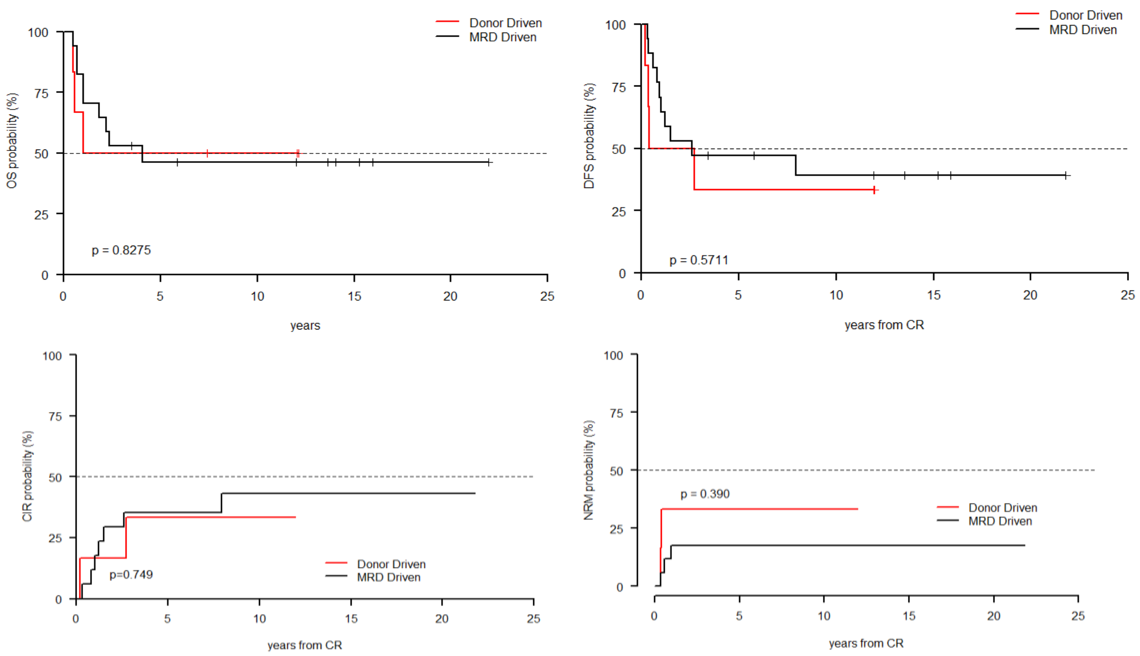

Figure S3. Impact of MRD-driven strategy on MRD negative patients belonging to the Intermediate risk category. We did not observe any difference in OS, DFS, CIR and NRM .

Table S1. Clinical characteristics of MRD-driven cohort patients (115) according to FCM-MRD level (Positive vs Negative).

	level	MRD-	MRD+	p
**n**		46	67	
**Sex (%)**	female	19 (41.3)	27 (40.3)	1.000
	male	27 (58.7)	40 (59.7)	
**Age (median [range])**		45.00 [21.00, 61.00]	43.00 [18.00, 64.00]	0.842
**WBC count (%)**	WBC count < 50 x 10^9/L	40 (87.0)	48 (71.6)	0.090
	WBC count > 50 x 10^9/L	6 (13.0)	19 (28.4)	
**FLT3-ITD (%)**	FLT3 negative	34 (91.9)	41 (64.1)	0.004
	FLT3 positive	3 (8.1)	23 (35.9)	
**NPM1-mutated (%)**	NPM negative	35 (89.7)	39 (63.9)	0.008
	NPM positive	4 (10.3)	22 (36.1)	
**Karyotype (%)**	Good	17 (40.5)	6 (9.2)	<0.001
	Intermediate	25 (59.5)	51 (78.5)	
	Poor	0 (0.0)	8 (12.3)	

Table S2. Results of OS, EFS, CIR, and NRM according to FCM-MRD level in favorable and intermediate cytogenetic risk groups (LR, IR). The analysis was not carried on adverse risk patients because a few tested MRD negative.

	MRD status	*10-yrs OS (%)*	*p-value*	*10-yrs DFS (%)*	*p-value*	*10-yrs CIR (%)*	*p-value*	*10-yrs NRM (%)*	*p-value*
Favorable-risk	*Negative*	65.5	0.180	**55.7**	**0.0003**	30.3	0.578	22.1	0.319
	Positive	50.3		**44.1**		38.2		8.4	
Intermediate-risk	*Negative*	54.8	0.071	**47.8**	**0.016**	**30.3**	**0.0003**	22.0	0.320
	Positive	31.6		**25.2**		**65.2**		8.4	
